# Pleckstrin-2 as a Prognostic Factor and Mediator of Gastric Cancer Progression

**DOI:** 10.1155/2021/5527387

**Published:** 2021-08-04

**Authors:** Jun Wang, Zhigang He, Bo Sun, Wenhai Huang, Jianbin Xiang, Zongyou Chen, Zhenyang Li, Xiaodong Gu

**Affiliations:** ^1^Department of General Surgery, Huashan Hospital, Fudan University, Shanghai, China; ^2^Department of General Surgery, Shanghai Songjiang District Central Hospital, Shanghai, China; ^3^Department of Gastric Surgery, Fudan University Shanghai Cancer Center, Shanghai, China

## Abstract

Pleckstrin-2 (PLEK2) is a crucial mediator of cytoskeletal reorganization. However, the potential roles of PLEK2 in gastric cancer are still unknown. PLEK2 expression in gastric cancer was examined by western blotting and real-time PCR. Survival analysis was utilized to test the clinical impacts of the levels of PLEK2 in gastric cancer patients. In vitro and in vivo studies were used to estimate the potential roles played by PLEK2 in modulating gastric cancer proliferation, self-renewal, and tumourigenicity. Bioinformatics approaches were used to monitor the effect of PLEK2 on epithelial-mesenchymal transition (EMT) signalling pathways. PLEK2 expression was significantly upregulated in gastric cancer as compared with nontumour samples. Kaplan-Meier plotter analysis revealed that gastric cancer patients with higher PLEK2 levels had substantially poorer overall survival compared with gastric cancer patients with lower PLEK2 levels. The upregulation or downregulation of PLEK2 in gastric cancer cell lines effectively enhanced or inhibited cell proliferation and proinvasive behaviour, respectively. Additionally, we also found that PLEK2 enhanced EMT through downregulating E-cadherin expression and upregulating Vimentin expression. Our findings demonstrated that PLEK2 plays a potential role in gastric cancer and may be a novel therapeutic target for gastric cancer.

## 1. Introduction

Gastric cancer remains a critical cancer; the incidence rate of gastric cancer ranks the fifth globally [1]. Although the diagnosis and management of gastric cancer have improved, the overall survival rate has not increased remarkably [2, 3]. The pronounced tendency towards recurrence and metastasis is the primary reason for the high rates of death and poor outcomes. The potential pathways and molecular mechanisms underlying gastric cancer development are still not fully elucidated [4, 5].

The human pleckstrin-2 (PLEK2) gene is located on chromosome 14q24.1 [6]. PLEK2 encompasses two conserved pleckstrin homology (PH) domains and an intervening disheveled-Egl-10-pleckstrin (DEP) domain. The expression of PLEK2 was found in various adherent cell lines [7]. PLEK2 promotes the shape changes associated with actin rearrangement. To date, the expression of PLEK2 and its role in gastric cancer progression and pathogenesis are still elusive.

In this study, PLEK2 expression was evaluated in The Cancer Genome Atlas (TCGA) datasets [8] and clinical gastric cancer samples using real-time PCR, western blotting, and immunohistochemistry (IHC). We detected that PLEK2 overexpression in gastric cancer was associated with shorter survival time. Our research also revealed the key functions of PLEK2 in promoting gastric cancer epithelial-mesenchymal transition (EMT), which may have implications for gastric cancer metastasis. Overall, our findings indicate that PLEK2 is a potential prognostic indicator and employed by self-renewal and proliferation which may provide a better understanding of gastric cancer tumourigenesis.

## 2. Materials and Methods

### 2.1. Patient Samples and Clinical Information

Gastric cancer tissue samples and matched nontumour samples were collected from the Fudan University Affiliated Huashan Hospital (Shanghai, China). Data from one hundred eight patients with pathologically proven gastric cancer were collected between May 2011 and April 2016. All patients who gave informed consent underwent pathological and radiological examinations to confirm the diagnosis. Data on the clinical characteristics of all participants were collected and made available. The therapies that participants underwent after enrollment were assessed, and follow-ups were performed until April 30, 2016. This research was authorized by the Ethics Committee at Fudan University-Affiliated Huashan Hospital.

### 2.2. Cell Cultures

The human gastric cancer cell lines BGC-823, MGC-803, MKN45, SGC-7901, AGS, and human embryonic kidney (HEK) 293T cells were obtained from Cobioer Biosciences Company (Nanjing, China). Short Tandem Repeat (STR) analysis was performed to authenticate all the cell lines before starting the study. The human gastric adenocarcinoma cells were cultured in RPMI-1640 medium containing 10% fetal bovine serum (Gibco, Carlsbad, CA, USA) and 1% penicillin-streptomycin. HEK 293T cells were maintained in DMEM (Gibco).

### 2.3. Vector Construction and Retroviral Infection

The wild-type PLEK2-CDS was PCR-amplified from HEK-293T cDNA using the following primers: forward 5′-TCGGAGCTGCTTCCTGGG-3′ and reverse 5′-CATGTTAGCTTTTTGATAGCTTCA-3′. Then, it was cloned into the pcDNA3.1/myc-His B vector (Invitrogen, Grand Island, NY, USA). The shRNAs targeting human PLEK2 (5′-AAGTGGCACGGTGGTGAAACA-3′) were cloned into pSilencer 4.1-CMV puro vectors (Ambion, Foster City, CA, USA). Retroviral production and infection were performed as the standard procedure. Stable cell lines expressing PLEK2 or shPLEK2 were selected for 10 days with 400 mg/mL G418 or 0.5 mg/mL puromycin, respectively.

### 2.4. Real-Time PCR

Total RNA from gastric cancer tissues and cells was isolated using the TRIzol reagent (Invitrogen, Carlsbad, CA, USA). Reverse transcriptase reaction was then performed using M-MLV reverse transcriptase (Takara, Dalian, China). All experiments were conducted following the manufacturer's instructions. Quantitative real-time PCR was performed using the CFX Connect Real-time PCR System (Bio-Rad, Hercules, CA, USA). GAPDH was used as the internal standard gene. The primers used for PCR were PLEK2-forward 5′-AGCCTGAGCACTGTGGAGTT-3′ and PLEK2-reverse 5′-GCTGCTGGCCTGAATGTAAT-3′. The primers used for PCR were GAPDH-forward 5′-ATGGGGAAGGTGAAGGTCG-3′ and GAPDH-reverse 5′-CTCCACGACGTACTCAGCG-3′.

### 2.5. Western Blotting

To prepare protein samples, cells washed with ice-cold phosphate-buffered saline (PBS) were lysed with a radioimmunoprecipitation assay (RIPA) buffer supplemented with Complete Protease Inhibitor. Protein concentration was determined using the BCA Protein Assay Kit (Beyotime, Haimen, China). Protein prepared from tissues and cells was loaded and separated with a 10% SDS polyacrylamide gel and electrotransferred to PVDF membranes (Millipore, Bedford, MA, USA). After blocking with 5% skim milk for 2 h, the membrane was incubated with primary antibodies overnight at 4°C, including an anti-GAPDH antibody (Proteintech, Rosemont, IL, USA), anti-PLEK2 antibody (Proteintech), anti-E-cadherin antibody (Cell Signaling Technology, Danvers, MA, USA), and anti-Vimentin antibody (Cell Signaling Technology). After washing with PBS, the membrane was incubated with secondary antibodies. Signals were detected with the enhanced ECL western blotting reagent (Millipore).

### 2.6. Immunohistochemistry

IHC was performed on tissues and adjacent nontumour tissues in 108 cases of gastric cancer, as recently described [9].

### 2.7. MTT Assays

A total of 5 × 10^3^ cells in 100 *μ*l of culture medium were plated in 96-well plates. The experiments were performed in triplicate. After 72 h, the cells were stained with 20 *μ*l of MTT dye (5 mg/ml, Sigma-Aldrich) for an additional 3-4 h at 37°C before the removal of MTT and the addition of 200 *μ*l of dimethyl sulfoxide (DMSO) (Sigma-Aldrich). The absorbance was measured at 570 nm (with a reference at 630 nm).

### 2.8. Colony Formation Assays

Cells were seeded and cultured on 6-well plates (200 cells/well) for 3 weeks. First, colonies were stained with 4% paraformaldehyde for 10 minutes and then fixed with 1% crystal violet (Beyotime) for 5 minutes. Colonies with greater than 50 cells were counted randomly.

### 2.9. Migration Assays

A Transwell membrane (6.5 mm diameter with 8 *μ*m pores, Corning) was used for the Transwell migration assay. The upper chambers were filled with RPMI 1640 medium with cells (2.5 × 10^5^ cells per well), and the lower chambers were loaded with RPMI 1640 containing 10% FBS to induce cell migration. After incubation for 16 h, cells on the top surface of the inserts were removed and cells that migrated to the bottom surface of the inserts were fixed and stained with 0.1% crystal violet for 30 minutes. Cells were counted in five random fields at ×200 magnification.

### 2.10. Wound Healing Assays

Cells seeded on 6-well plates were cultured until confluence. A homogeneous wound was scratched with a yellow pipette tip. Three wounds were made for each sample. Images of cells migrating into the wound were photographed and measured at zero time and after 24 h.

### 2.11. Animal Model

Mice were randomly assigned to experimental groups for the animal experiments. MGC803 cells (2 × 10^6^) transduced with lentiviruses expressing shPLEK2 or PLEK2 were harvested and resuspended in sterile PBS. Groups of 4-week-old BALB/c nu/nu female mice were subcutaneously injected with the above cell suspensions. Tumour development was detected after 5 days. The formula (*a* × *b*^2^)/2 was used to monitor the tumour volume (*a* is the length and *b* is the width of the tumour). Twenty-one days later, all mice and controls were humanely sacrificed, and xenograft tumours were excised and measured. The animal study was reviewed and approved by the Animal Care and Use Committee of Fudan University.

### 2.12. Statistical Analysis

All data are presented using SPSS 22.0 (SPSS Inc., Chicago, IL, USA). Data are presented as mean ± standard deviation. For experiments involving three or more groups, comparisons between groups for statistical significance were performed with 2-tailed paired Student's *t*-tests. The correlations of PLEK2 with clinicopathological characteristics were examined using the chi-square test. The Kaplan-Meier method was used to calculate survival. Kaplan-Meier survival curves were generated using GraphPad Prism 5, and the *P* values were calculated using the logrank test. A Cox proportional hazards model was used to calculate the adjusted hazard ratio (HR) with a 95% confidential interval (CI). All statistical tests were two-sided. A *P* value of <0.05 was considered significant.

## 3. Results

### 3.1. PLEK2 Was Upregulated in Human Gastric Cancer

**Figure 1:**
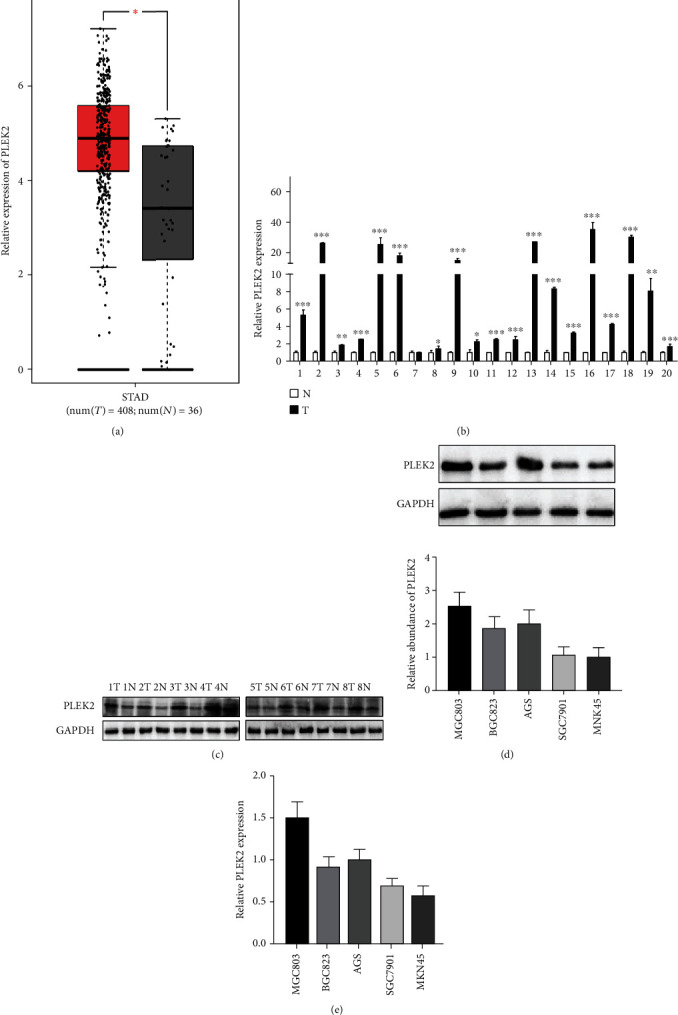
PLEK2 expression was elevated in gastric cancer. (a) The expression of PLEK2 mRNA in primary gastric cancer and adjacent noncancerous tissues in TCGA dataset. (b) The expression of PLEK2 mRNA in primary gastric cancer and adjacent noncancerous tissues by real-time PCR. The expression levels were normalized to those of GAPDH. (c) Representative western blots demonstrating the expression of PLEK2 protein in each of the primary gastric cancer and adjacent noncancerous tissues paired from the same patient. The expression levels were normalized to those of GAPDH. (d) The expression of PLEK2 protein in gastric cancer cell lines. GAPDH was the loading control. (e) Relative PLEK2 mRNA expression in gastric cancer cell lines. STAD: stomach adenocarcinoma; num: number; T: tumour; N: noncancerous tissues.

To investigate the expression patterns of PLEK2 in gastric cancer, we first monitored PLEK2 expression in TCGA database in GEPIA, TCGA database-driven web portal for gene expression profiling and interactive analysis (http://gepia.cancer-pku.cn/). We found that PLEK2 was remarkably upregulated in the RNA-seq dataset of 444 gastric cancer samples (Figure 1(a)). Real-time PCR analysis showed that PLEK2 mRNA expression was increased in 19 of 20 (95%) cases of cancer tissues. The relative abundance of PLEK2 mRNA expression was up to 35-fold higher in all examined samples (Figure 1(b)). Western blotting illustrated that the expression of PLEK2 was stronger in gastric cancer tissues than in the peritumoural gastric tissues (Figure 1(c)). Western blotting and real-time PCR analysis also revealed that gastric cancer cell lines, including BGC-823, MGC-803, MKN45, SGC-7901, and AGS, exhibited high PLEK2 protein and mRNA expression (Figures 1(d) and 1(e)).

### 3.2. Increased PLEK2 Was Associated with Poor Survival in Gastric Cancer

**Figure 2:**
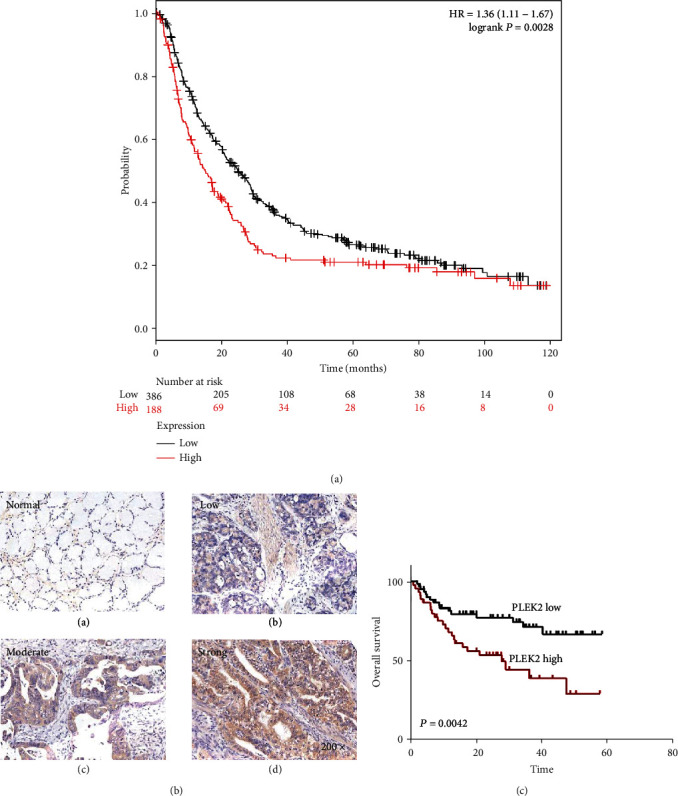
Survival analysis of patients with gastric cancer according to the expression of PLEK2. (a) The effect of PLEK2 on overall survival using 574 cancer samples based on the Kaplan-Meier plotter. (b) Examples of gastric cancer tissues immunostained for PLEK2. Different PLEK2 staining intensities are exemplified. (c) Kaplan-Meier analysis of overall survival in patients with gastric cancer (*n* = 108, *P* = 0.0042). HR: hazard ratio.

We further determined the relationship between PLEK2 mRNA and the overall survival of gastric cancer patients based on the web-based database Kaplan-Meier plotter. A total of 574 patients were included from the GSE14210, GSE15459, GSE22377, GSE29272, and GSE51105 datasets. Kaplan-Meier plotter analysis revealed that those with higher PLEK2 levels had substantially poorer overall survival (HR: 1.36, 95% CI: 1.11–1.67, *P* = 0.0028) compared with those with lower PLEK2 levels (Figure 2(a)).

Then, we evaluated the expression of PLEK2 by IHC staining in clinical gastric cancer tissues and found high expression in 46 cases (42.6%) and low expression in the other 62 cases (57.4%). PLEK2 showed no signal or a weak signal in peritumoural gastric tissues (Figure 2(b)). A chi-square test demonstrated that PLEK2 protein was prominently connected with the tumour grade (*P* = 0.025), tumour size (*P* = 0.033), lymph node invasion (*P* = 0.045), distant metastasis (*P* = 0.011), and advanced TNM stage (*P* = 0.039) (Table 1). Kaplan-Meier survival analysis also showed that high PLEK2 levels were closely related to poor overall survival (Figure 2(c)).

### 3.3. Decreased PLEK2 Repressed the Proliferation and Migration of Gastric Cancer Cells

**Figure 3:**
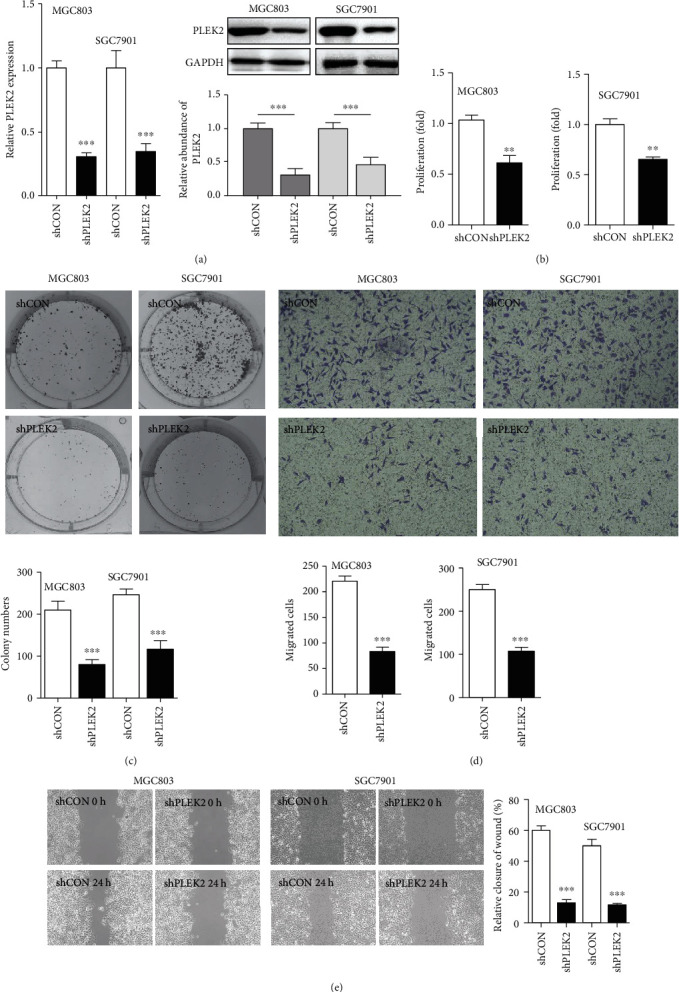
Knockdown of PLEK2 significantly suppressed gastric cancer cell proliferation, colony formation, and migration. (a) The stable knockdown of PLEK2 in MGC803 and SGC7901 cells by shRNA sequences (shPLEK2). The knockdown effect was verified at the mRNA and protein levels. (b) PLEK2 knockdown significantly reduced the proliferation rate of MGC803 and SGC7901 cells. (c) The knockdown of PLEK2 impaired the colony formation ability of gastric cancer cells. (d) The knockdown of PLEK2 suppressed gastric cancer cell migration. (e) The knockdown of PLEK2 significantly reduced the wound healing ability of gastric cancer cells. ^∗^*P* < 0.05, ^∗∗^*P* < 0.01, and ^∗∗∗^*P* < 0.001 (*t*-test).

To explore the effect of PLEK2 in human gastric cancer, we established stable PLEK2 knockdown models in MGC803 and SGC7901 cells by expressing short hairpin RNAs (shPLEK2). The knockdown of PLEK2 resulted in a significant reduction in the expression of PLEK2 RNA and protein levels, as revealed by real-time PCR and western blotting (Figure 3(a)). The downregulation of PLEK2 resulted in a remarkable decrease in cell proliferation, as shown by MTT assays (Figure 3(b)). The knockdown of PLEK2 significantly inhibited gastric cancer cell colony-forming abilities (Figure 3(c)). PLEK2 knockdown led to a significant decrease in cell migratory abilities (Figure 3(d)). Similarly, PLEK2 knockdown cells exhibited slower migration in the wound healing assays (Figure 3(e)). These results indicated that PLEK2 improves the self-renewal and migratory capacity of gastric cancer cells.

### 3.4. Overexpression of PLEK2 Promoted Gastric Cancer Proliferation and Migration

**Figure 4:**
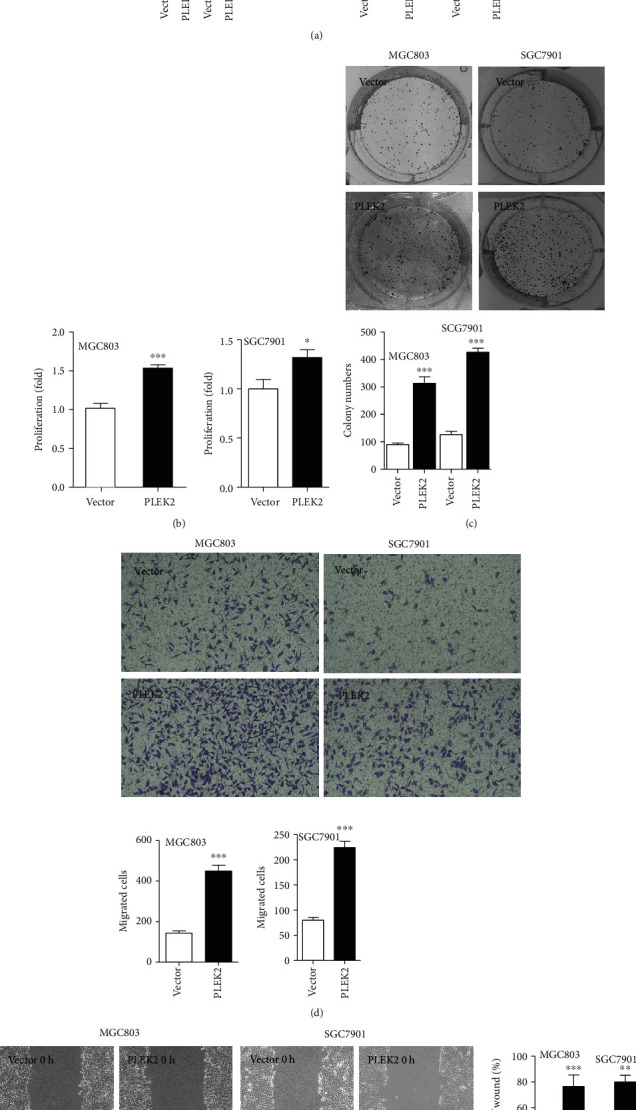
Overexpression of PLEK2 promoted gastric cancer proliferation, colony formation, and cell migration. (a) The overexpression of endogenous PLEK2 was confirmed at the mRNA and protein levels. (b) The overexpression of PLEK2 increased the cell proliferation rate in MGC803 and SGC7901 cells. (c) The overexpression of PLEK2 enhanced the colony formation ability of gastric cancer cells. (d) The overexpression of PLEK2 accelerated gastric cancer cell migration. (e) The overexpression of PLEK2 enhanced gastric cancer cell wound healing ability. ^∗^*P* < 0.05, ^∗∗^*P* < 0.01, and ^∗∗∗^*P* < 0.001 (*t*-test).

We upregulated PLEK2 expression in gastric cancer cells to determine its effects. The upregulation of endogenous PLEK2 in MGC803 and SGC7901 cells was confirmed at both the mRNA and protein levels (Figure 4(a)). As expected, the upregulation of PLEK2 accelerated gastric cancer cell proliferation in the MTT assay (Figure 4(b)). The overexpression of PLEK2 significantly promoted gastric cancer cell colony-forming abilities (Figure 4(c)). The overexpression of PLEK2 significantly improved gastric cancer cell migration in a Transwell assay (Figure 4(d)). Similarly, PLEK2-overexpressing cells more quickly migrated to fill in space in wound healing assays (Figure 4(e)).

### 3.5. Inhibition of PLEK2 Effectively Suppresses Tumour Growth in Nude Mice

**Figure 5:**
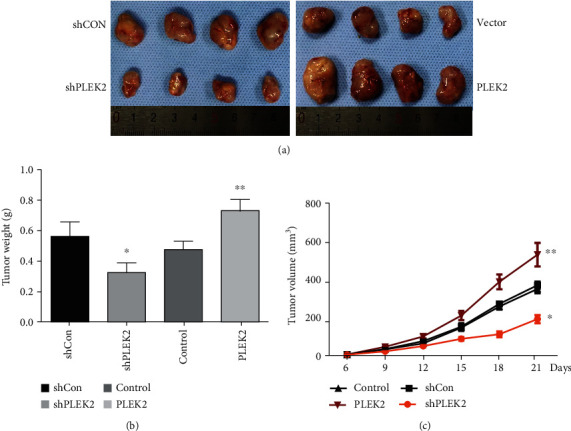
PLEK2 influenced gastric cancer tumour growth in a nude mouse model. (a) The inhibition of PLEK2 impaired gastric cancer tumour growth in a nude mouse model (left), while the overexpression of PLEK2 promoted gastric cancer tumour growth (right). The size of the tumour formed in the subcutaneous implantation mouse model was monitored every three days. (b) The mean tumour weights of each group. (c) Growth curves for tumour volumes at the indicated times.

We employed a nude mouse tumourigenicity assay to detect the functional roles of PLEK2 in gastric cancer tumourigenicity. Stable PLEK2-overexpressing MGC803 cells showed remarkably improved tumour growth and tumour weight compared with those observed in the control groups. Conversely, the knockdown of PLEK2 significantly suppressed tumour growth and weight compared with the level of suppression in the nontarget shRNA control groups (Figure 5(a)). In addition, there was a difference in the size and weight of the tumours over time in the PLEK2 upregulation and downregulation groups (Figures 5(b) and 5(c)). Collectively, the results showed that PLEK2 may serve as an oncogene that enhances gastric cancer proliferation.

### 3.6. PLEK2 Participates in EMT

**Figure 6:**
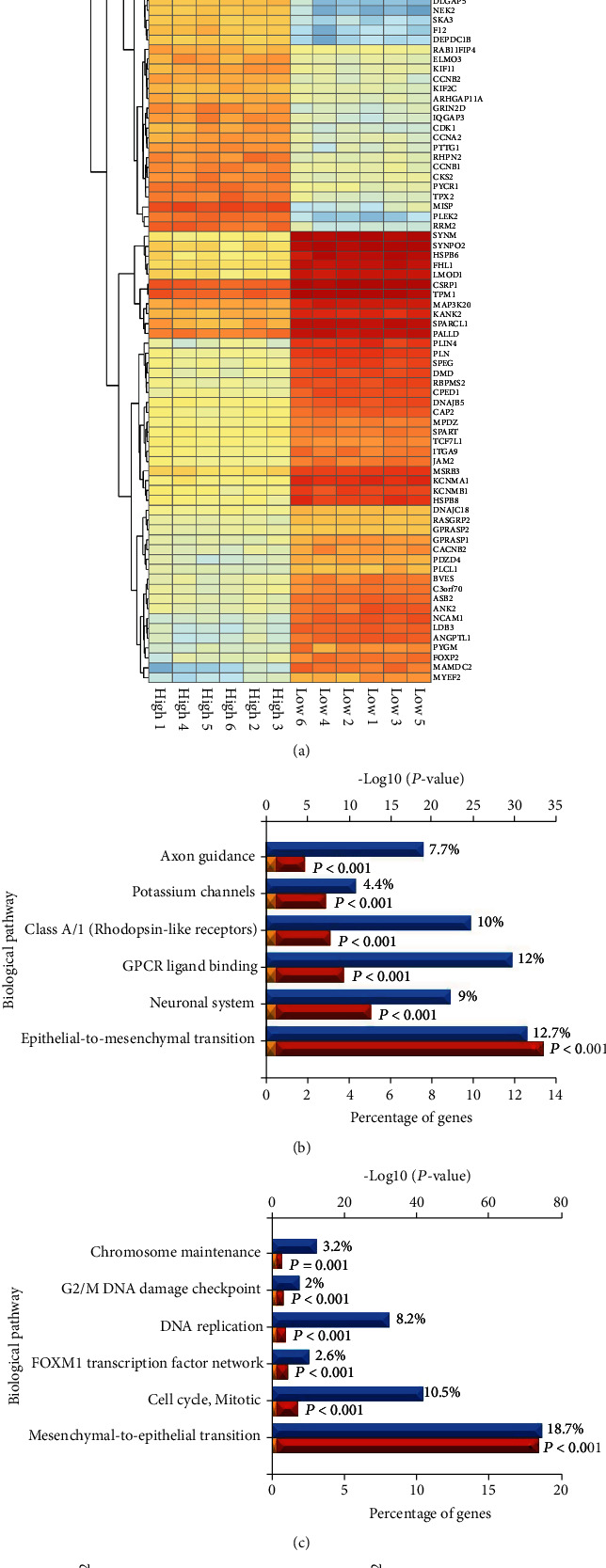
PLEK2 promotes EMT in gastric cancer cells. (a) The heat map shows the top 100 differentially expressed genes related to PLEK2. (b) Biological pathways enriched in genes that are more than 2-fold abundant in the PLEK2-high group compared to the PLEK2-low group. (c) Biological pathways enriched in genes that are more than 2-fold deficient in the PLEK2-high group compared to the PLEK2-low group. (d) PLEK2 overexpression induces hallmarks of EMT in gastric cancer cells, while PLEK2 knockdown inhibits EMT.

To reveal the potential mechanisms of PLEK2-mediated cell invasion and proliferation, we used TCGA database to compare the gene expression profiles of the 6 PLEK2-highest and the 6 PLEK2-lowest databases. The top 100 differentially expressed genes are shown on the heat map in Figure 6(a). We further performed functional enrichment analysis using FunRich software. The biological pathways enriched in genes that are more than 2-fold abundant in the PLEK2-high group compared to the PLEK2-low group are displayed in Figure 6(b), and the biological pathways enriched in genes that are more than 2-fold deficient are shown in Figure 6(c). Most of the identified genes are already known to be involved in EMT. As the most significant pathways are related to EMT, we detected the level of Vimentin and E-cadherin in gastric cells after PLEK2 upregulation and downregulation. As shown in Figure 6(d), the knockdown of PLEK2 showed enhanced epithelial marker (E-cadherin) expression and decreased mesenchymal marker (Vimentin) expression. PLEK2 overexpression inhibited E-cadherin expression and induced Vimentin expression. The results showed that PLEK2 promoted EMT in gastric cancer cells.

## 4. Discussion

PLEK2 encompasses two PH domains and an intervening DEP domain, which control cytoskeletal reorganization in various cells. The DEP domain is in the center, and the two PH domains are in the amino- and carboxyl-terminals, which are important for PLEK2 colocalization with the actin cytoskeleton at the immune synapse and integrin clusters and involve a broad range of cellular functions [10–13]. Previous studies have shown that PLEK2 regulates actin dynamics and cofilin's mitochondrial localization during erythropoiesis [14, 15]. PLEK2 induces lamellipodia formation depending on its DEP domain. The DEP domain mutant disrupts the formation of lamellipodia and membrane ruffles [16, 17]. Unlike PLEK1, which is restricted to immune cells, PLEK2 has been identified in various adherent cell lines involved in cellular signalling and cytoskeleton organization [18, 19]. A study reported that PLEK2 is vital for the modulation of the actin cytoskeleton, cellular transdifferentiation, and prosurvival in the early stage of terminal erythroblasts and prevents early-stage terminal erythroblasts from oxidative damage [14, 20]. An important effect of PLEK2 in lineages of haematopoietic cells was discovered by modulating cytoskeleton organization and cell apoptosis through PLEK2's interplay with members of Rac1 signalling, such as cofilin. In another report, PLEK2 was found to be an effector of the JAK2/STAT5 pathway and an important regulator in the pathogenesis of JAK2V617F-induced myeloproliferative neoplasms, suggesting that PLEK2 is a feasible therapeutic target of myeloproliferative neoplasms [21]. Bach et al.'s group demonstrated that PLEK2, as an effector of PI3K, increases Jurkat cell spreading through both *α*4*β*1 and the T-cell receptor [22]. However, the biological functions of PLEK2 in tumour progression have not been well defined, especially in gastric tumourigenesis.

Recent studies have found that pancreatic cancer tissues exhibited an enhancement of PLEK2 expression [23]. PLEK2 could promote the self-renewal and proliferation of pancreatic cancer stem cells. PLEK2 has been found to be overexpressed in the blood of melanoma patients in all stages of disease, suggesting its potential function as a liquid biopsy marker for early diagnosis [24]. PLEK2 also mediated vascular invasion and metastasis in non-small-cell lung cancer and gallbladder cancer [25, 26]. In this research, we studied the effect of PLEK2 in gastric cancer utilizing survival analysis, cell test in vitro, and nude mouse experiment in vivo. PLEK2 upregulation in gastric cancer cells increased cell migration, invasion, proliferation, and self-renewal in vitro and tumourigenesis in vivo, indicating that PLEK2 may have an oncogenic function in gastric cancer.

Now, bioinformatics tools are becoming important. Yang et al.'s group performed a comprehensive bioinformatics analysis on microarray data of myeloma cells. PLEK2 was identified as a potential therapeutic target [27]. In this study, we utilized FunRich to analyse the biological pathways related to PLEK2. The results revealed that the most significant biological pathway was EMT or mesenchymal-epithelial transition (MET). Further analysis confirmed the role of PLEK2 in promoting EMT. Previous studies reported that PLEK2 mRNA was upregulated in non-small-cell lung cancer cells with TGF-*β*-induced EMT, demonstrating the crucial role of PLEK2 in tumour invasion [25, 28]. We found that PLEK2 was preferentially accumulated in gastric cancer tissues and probably enhanced the EMT mechanism.

PLEK2 participates in actin reorganization in a PI3-kinase-dependent manner. PLEK2 has been previously reported to promote the migration of HCC2998 and COS-1 cells and T lymphocytes by binding with PI3K [20, 22]. Mounting evidences suggest that cytoskeleton reconstruction plays the crucial role in the EMT process. The dynamic reorganization of the actin cytoskeleton is a prerequisite for the morphology and invasive behaviour of cancer cells. In this study, we confirmed the role of PLEK2 in promoting EMT in gastric cancer cells. Further researches are needed to elucidate the mechanism underlying the role that PLEK2 plays in EMT regulation during cancer progression.

In summary, we discovered that PLEK2 was remarkably elevated in gastric cancer. Its upregulation functionally promoted the aggressiveness of gastric cancer cells by driving EMT. Our work suggests that targeting PLEK2 might be an effective therapeutic strategy to treat PLEK2-high gastric cancer.

## Figures and Tables

**Table 1:** Relationships between PLEK2 expression and clinicopathological characteristics.

Characteristics	PLEK2 expression		*P* value
	Low	High	
Age (years)			
<65	29	25	
≥65	33	21	0.436
Gender			
Male	24	20	
Female	38	26	0.618
Grade			
Well or moderate	54	32	
Poor	8	14	**0.025**
Pathologic stage			
I-II	34	16	
III-IV	28	30	**0.039**
T stage			
T1-T2	24	9	
T3-T4	38	37	**0.033**
N stage			
N0	35	17	
N1-N3	27	29	**0.045**
Distant metastasis			
M0	57	34	
M1	5	12	**0.011**

*P* values in bold indicate significant results.

## Data Availability

The data used to support the findings of this study are available from the corresponding author upon request.
